# Megadepth: efficient coverage quantification for BigWigs and BAMs

**DOI:** 10.1093/bioinformatics/btab152

**Published:** 2021-03-08

**Authors:** Christopher Wilks, Omar Ahmed, Daniel N Baker, David Zhang, Leonardo Collado-Torres, Ben Langmead

**Affiliations:** Department of Computer Science, Johns Hopkins University, Baltimore, MD 21218, USA; Department of Computer Science, Johns Hopkins University, Baltimore, MD 21218, USA; Department of Computer Science, Johns Hopkins University, Baltimore, MD 21218, USA; Department of Molecular Neuroscience Institute of Neurology, University College London (UCL), London WC1E 6BT, UK; NIHR Great Ormond Street Hospital Biomedical Research Centre, University College London, London WC1E 6BT, UK; Genetics and Genomic Medicine, Great Ormond Street Institute of Child Health University College London, London WC1E 6BT, UK; Lieber Institute for Brain Development, Baltimore, MD 21205, USA; Department of Computer Science, Johns Hopkins University, Baltimore, MD 21218, USA

## Abstract

**Motivation:**

A common way to summarize sequencing datasets is to quantify data lying within genes or other genomic intervals. This can be slow and can require different tools for different input file types.

**Results:**

Megadepth is a fast tool for quantifying alignments and coverage for BigWig and BAM/CRAM input files, using substantially less memory than the next-fastest competitor. Megadepth can summarize coverage within all disjoint intervals of the Gencode V35 gene annotation for more than 19 000 GTExV8 BigWig files in approximately 1 h using 32 threads. Megadepth is available both as a command-line tool and as an R/Bioconductor package providing much faster quantification compared to the rtracklayer package.

**Availability and implementation:**

https://github.com/ChristopherWilks/megadepth, https://bioconductor.org/packages/megadepth.

**Supplementary information:**

Supplementary data are available at *Bioinformatics* online.

## 1 Introduction

Many sequencing data analyses are concerned with the depth of coverage in genomic regions. For example, RNA-seq alignments are often quantified within annotated intervals. Other examples include copy-number analysis of DNA-seq data or quantification of coverage under ChIP-seq peaks. The need is particularly pronounced for RNA-seq, where datasets may need periodic requantification with respect to updated or alternative gene annotations (Collado-Torres et al., 2017).

BAM files store read alignments in a compressed and indexed form allowing random access (Li et al., 2009). CRAM files are similar, additionally using reference-based compression (Hsi-Yang Fritz et al., 2011). BigWig files (Kent et al., 2010) store coverage vectors (not alignments) in a compressed and indexed form. While BAM and CRAM contain more information than BigWigs, BigWigs are also used for long-term storage because they are much smaller—often by an order of magnitude—while keeping enough information for requantification.

Mosdepth (Pedersen et al., 2018) is an efficient quantification tool designed for BAM/CRAM files that can summarize coverage within intervals or across the entire file. Samtools and Sambamba (Li *et al.*, 2009; Tarasov et al., 2015) can extract coverage from genomic regions within BAM and other related files (e.g. BED, VCF), though they cannot summarize coverage (e.g. sum or average). WiggleTools (Zerbino et al., 2014) and bwtool (Pohl et al., 2014) can extract and summarize coverage from both BigWig and BAM/CRAM files, and pyBigWig (Ramírez et al., 2016) is a Python module with similar functionality. rtracklayer is an R/Biconductor package that handles both BAM and BigWig formats. In contrast, Megadepth supports BAM, CRAM and BigWig inputs. It is faster while providing more features than other tools.

## 2 Methods

Megadepth processes BAMs one chromosome at a time, allocating a chromosome-length array in memory. It scans alignments in the BAM—possibly looking only within user-specified regions—and tallies base coverage in the array, either via the increment/decrement approach (Pedersen *et al.*, 2018; Wiewiórka et al., 2019) or by storing explicit counts, depending on the operation. Megadepth uses the same general approach for BigWig files, scanning them base-by-base. Megadepth can output per-base coverage counts from BAM/CRAM inputs in a BED or BigWig file. Besides base-level coverage, Megadepth can additionally output per-interval coverage sums or averages as a BED file and an overall area-under-coverage statistic. Megadepth can be configured to use multiple HTSlib threads for reading BAMs, speeding up block-gzip decompression (Supplementary Note 1). Since Megadepth’s single-threaded processing of BigWigs is already extremely fast (typical files take seconds) multi-threading is not implemented for that mode (Supplementary Note 2). Megadepth can query remote BAM, CRAM and BigWig files via an HTTP or FTP URL. This remote-access functionality, built into the htslib and libBigWig libraries and typically using libCurl, leverages the partitioned nature of all three formats, requesting byte ranges of the remote file. Megadepth is written in C++11 and utilizes the HTSLib (v1.11) and libBigWig (v0.4.4) (Ramírez *et al.*, 2016) libraries. Binaries are available for Linux x86-64, MacOS x86-64 and Windows x86-64.

## 3 Results

We used BigWig-enabled tools to compute coverage sums for 5.5 million repetitive-element intervals across 10 BigWig files from GTEx brains (upper half of Table 1). Megadepth was at least four times faster than all other tools while using 543 MiB of memory, the second lowest memory footprint among the five tools. WiggleTools was the next-fastest tool but it used ∼10 GiB of memory, limiting its utility on some systems. The megadepth-R package, which wraps Megadepth functionality for R, was 47 times faster and used a fraction of memory (808 MiB) compared to rtracklayer (∼14 GiB), the only R/Bioconductor tool we tested. We performed more comparisons using different BigWigs files and intervals sets, including disjoint intervals from Gencode V35 (Supplementary Note 3). Overall, Megadepth was the fastest tool, though the speed gap was smaller for smaller interval sets; e.g. WiggleTools was only 30% slower for the Gencode V35 set. In addition, we recently used Megadepth to re-quantify all disjoint intervals of the Gencode V35 gene annotation for 19,214 GTExV8 BigWig files in about 1 h using 32 threads.

**Table 1.** Top: Comparison of BigWig-enabled tools when computing coverage sums over repetitive-element intervals for 10 GTEx brain tissue BigWigs and Bottom: Comparison of BAM-enabled tools when computing coverage means over exome intervals for a 30× WGS BAM

Tool	Relative time	Run time	Memory (MiB)	BAM input	BigWig input	MacOS	Windows native	R interface
Megadepth (BigWig)	1.00	1 m:57 s	543	Yes	Yes	Yes	Yes	Yes
megadepth-R (BigWig)	2.13	4m:09 s	808	Yes	Yes	Yes	Yes	Yes
WiggleTools (BigWig)	4.06	7 m:54 s	10,379	Yes	Yes	Yes	No	No
pyBigWig	68.13	2 h:12 m:36 s	7	Yes	Yes	Yes	No	No
bwtool	90.48	2 h:56 m:06 s	750	No	Yes	No	No	No
rtracklayer	100.61	3 h:15 m:49 s	14,074	Yes	Yes	Yes	No	Yes
Megadepth (BAM)	1.00	2 m:17 s	1,016	Yes	Yes	Yes	Yes	Yes
Mosdepth	5.58	12 m:43 s	1,911	Yes	No	Yes	No	No
Samtools	40.05	1 h:31 m:20 s	15	Yes	No	Yes	Yes	Yes
Sambamba	3.55	8 m:05 s	157	Yes	No	Yes	No	No
WiggleTools (BAM)	628.56	23 h:55 m:13 s	372	Yes	Yes	Yes	No	No

*Note*: Each tool’s features are also summarized. All runs use a single thread except WiggleTools, which non-optionally uses extra threads for input and output.

Next we used the BAM-enabled tools to compute mean coverage within a set of 191 744 exome-capture intervals across a single 30× coverage whole-genome DNA-seq BAM (lower half of Table 1). Megadepth was at least three times faster than other tools. While Megadepth used more memory (∼1 GiB) compared to samtools, sambamba and WiggleTools, it used about half the memory of the next-fastest tool, Mosdepth. Megadepth BAM processing is generally slower than BigWig processing since BAM files store substantially more information, e.g. including read sequences and base qualities. Supplementary Note 4 describes comparisons on BAM and CRAM files where the tools are configured to output base-by-base coverage values. While Megadepth is still fastest, some of the differences are very small, e.g. Mosdepth is only 12% slower. But the difference grows when using an RNA-seq BAM file, where Mosdepth takes 2.7× the time. We also measured the time required to analyze an entire DNA-seq BAM file within 500 bp windows, similar to a benchmark in the Mosdepth study (Supplementary Note 5). Finally, we performed further BAM and CRAM benchmarks using query intervals (Supplementary Note 6).

We also compared the BAM-enabled tools’ performance when processing RNA-seq BAM files. We evaluated the tools when extracting all genomic bases (Supplementary Note 4 and Table 4b), when computing mean coverage over disjoint intervals from the Gencode v35 annotation (Supplementary Note 6 and Table 6c), and when computing mean coverage over a larger number of intervals corresponding to repetitive elements (Supplementary Note 6 and Table 6e). In all cases, Megadepth is the fastest tool. In the case of the repetitive element quantification, Mosdepth comes to within two times the speed of Megadepth. In the case where all bases are extracted, Wiggletools is competitive on speed (within two times), while also using less memory. Overall, Megadepth exhibited an advantageous mix of speed and memory efficiency, always achieving greater speed and lower memory footprint compared to Mosdepth, and always achieving greater speed—sometimes by orders of magnitude—compared to WiggleTools.

## 4 Discussion

Quantification is a common way to analyze new datasets and to re-analyze archived sequencing datasets (Collado-Torres *et al.*, 2017; Zhang et al., 2020). Megadepth further facilities this by providing an R/Bioconductor interface, readily used in combination with recount2 and other R-based resources. BigWig support is of particular import since BigWigs are much smaller than BAMs, while still containing the information needed to re-quantify. Megadepth provides this both by enabling rapid conversion from BAM to BigWig—a onetime cost—and by rapidly re-quantifying the resulting BigWig with respect to newer interval sets, possibly many times.

Finally, Megadepth supports extraction of alternate base coverage, junction co-occurrences, and fragment length distribution for paired samples (Supplementary Fig. S3). These functions were added for the purpose of a ‘one-time’ BAM processing tool which could extract and summarize multiple coverage-related statistics from an RNA-seq BAM after alignment so that the BAM could be discarded while much of the pertinent information is kept. Alternate base coverage can be potentially used for genotyping and allele-specific expression prediction. Junction co-occurrences provide contextual information for splice junctions which occur in a specific read or fragment alignment (either mate in a pair) which can be used to infer that these junctions are from the same transcript. Fragment length distributions contribute to statistical analyses of the sequence fragments derived from a sample where the BAM is no longer available.

## Funding

C.W., O.A., D.N.B. and B.L. were supported by National Institutes of Health/National Institute of General Medical Sciences (R01GM118568 to B.L.). L.C.T., B.L. and C.W. were supported by R01GM121459 to Dr Kasper Hansen. D.Z. was supported by UK Medical Research Council funding awarded to Dr Mina Ryten (Tenure Track Clinician Scientist Fellowship, MR/N008324/1).

*Conflict of Interest:* none declared.

## Supplementary Material

btab152_Supplementary_Data
