# Supplementary material for: Megadepth: efficient coverage quantification for BigWigs and BAMs
Source: Bioinformatics. 2021 Mar 8;37(18):3014–6. doi: 10.1093/bioinformatics/btab152 (PMC8528031; doi:10.1093/bioinformatics/btab152)
Supplement: btab152_Supplementary_Data [file btab152_supplementary_data.pdf]

Version Note
The versions of the software used throughout the benchmarks in this set of supplementary notes are:

- Megadepth 1.0.9 (using HTSLib 1.11 and libBigWig 0.4.4)
- Mosdepth 0.3.1 (statically compiled by these authors to use HTSLib 1.11)
- Sambamba 0.7.1
- Samtools 1.11
- WiggleTools 1.2.4
- pyBigWig 0.3.17
- bwtool 1.0
- rtracklayer 1.50.0

All benchmarks were run on a machine with four Intel Xeon E7-4830 v4 (Broadwell) 2.00 GHz processors (112 threads in total) and 1 TB of RAM. Experiments read from/wrote to a direct-attached storage array.

Unlike Megadepth and some of the other tools tested here, WiggleTools doesn't support a multithreaded option, but separate threads are used for certain of its input/output operations (e.g. reading from a BAM file). Therefore WiggleTools' performance profile can benefit from multiple threads in the following benchmarks even when the other tools are set to use a single thread, this behavior is not under control of the user.

Windows Native Version Note
In Table 1 of the main manuscript, the term "Windows Native" refers to a pre-compiled binary that does not require Windows Subsystem for Linux (WSL), Cygwin, or similar compatibility layer. Megadepth does not support writing BigWig files on Windows at this time.

Supplementary Note S1: Comparing megadepth and mosdepth's scalability with additional BAM decompression threads

Description: This experiment looks at time savings that occur when trying to compute mean coverage across regions on BAMs with each additional thread. This experiment does it for both an annotation consisting of 500 bp sliding windows and hg19 exome annotation.

Data:

- Genomic BAM:
  - Used ERR1019041.bam from Simons Genome Diversity Panel (Mallick et al., 2016)
  - Data was preprocessed with following commands:
    - samtools view -b -s 12.75 ERR1019041.bam > ERR1019041_sub.bam
    - samtools sort ERR1019041_sub.bam > ERR1019041_sorted.bam
    - samtools -b view chr1 … chr22 chrX chrY > ERR1019041_sorted_minusM.bam
    - samtools index ERR1019041_sorted.bam

    ○ For reference, this BAM is 69 GB
- Used hg19 exome from this link: https://www.twistbioscience.com/resources/bed-file/ngs-human-core-exome-panel-bed-files

Sub-Experiments:

Table S1a: Looking at time improvements with each additional BAM decompression thread when computing mean coverage of Genomic BAM file (ERR1019041) with sliding window regions of 500 bp

Format	Tool	Additional Threads	Command
BAM	megadepth	t = 0:8	megadepth <bam_file.bam> --threads <t> --gzip --op mean --annotation 500 --prefix <output_prefix> --no-annotation-stdout
BAM	mosdepth	t = 0:8	mosdepth -F 260 -n --by 500 --threads < t> <output_prefix> <bam_file.bam>

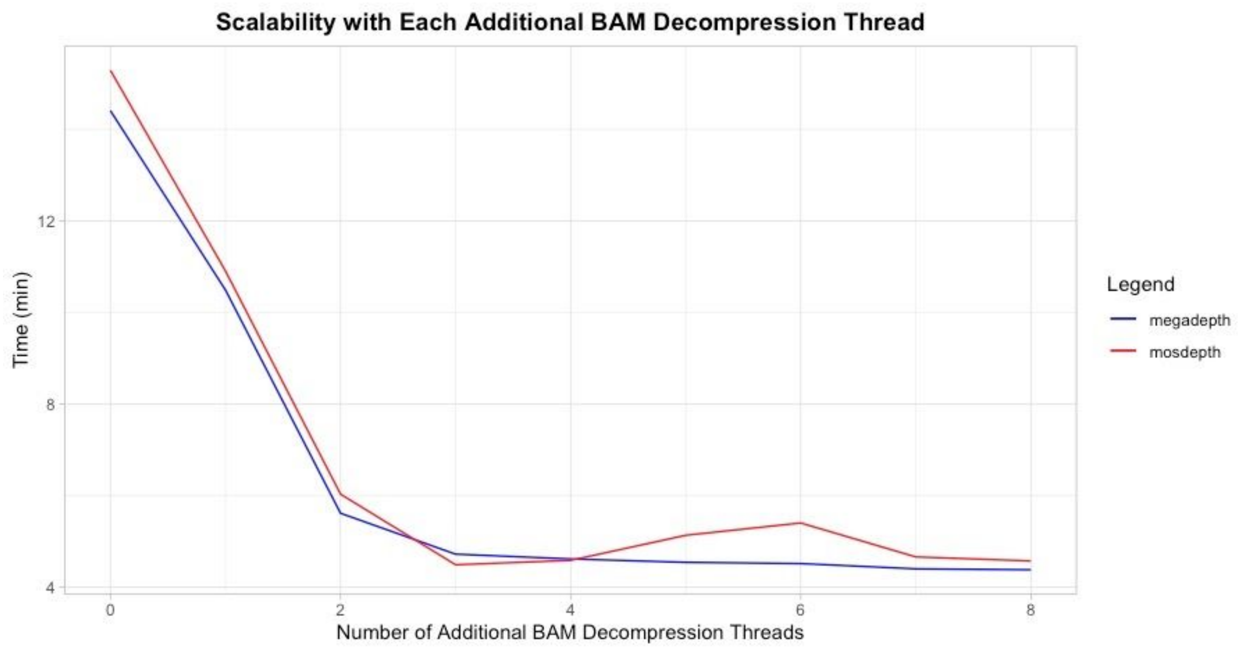

Figure S1. Thread scaling of whole-genome base coverage across a BAM

Table S1b: Looking at time improvements with each additional BAM decompression thread on computing mean on regions for Genomic BAM file (ERR1019041) with hg19 exome annotation

Format	Tool	Additional Threads	Command
BAM	megadepth	t = 0:8	megadepth <bam_file.bam> --threads < t> --gzip --op mean --annotation <bed_file.bed> --prefix <output_prefix> --no-annotation-stdout
BAM	mosdepth	t = 0:8	mosdepth -F 260 -n --by <bed_file.bed> --threads <t> <output_prefix> <bam_file.bam>

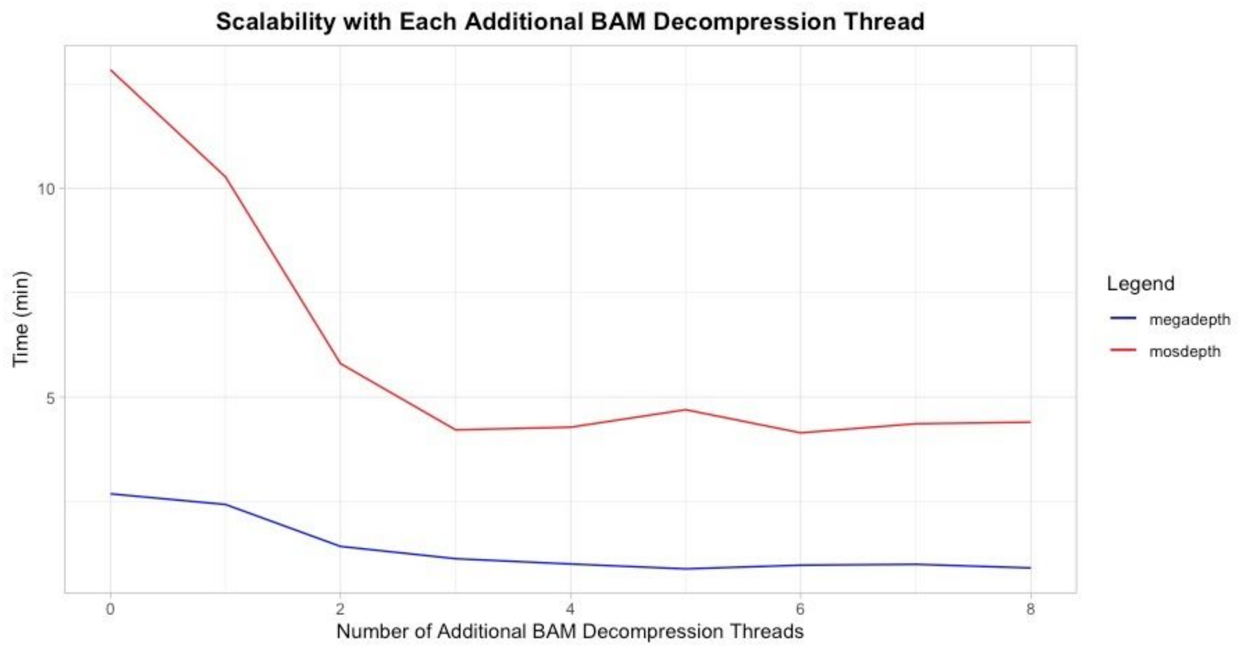

Figure S2. Thread scaling of exome base coverage across a BAM

Supplementary Note S2: Looking at megadepth's ability to process all the BigWigs produced from GTEx

Description: Examine the time and memory needed for megadepth to compute the sums on regions for all the BigWig files produced from GTEx*. We used the GNU parallel (Tange 2011) command to run multiple instances of Megadepth concurrently on the same system.

Data:

- Input data used was BigWig files produced from GTEx (19,214 BigWig files), available through recount3 project
- Annotation used was Gencodev35 (Frankish et al., 2019) disjoint exon

Sub-Experiments:

Table S2a: Examining time and memory used by megadepth to compute sum of regions on all GTEx BigWigs on GencodeV35 disjoint exon regions

Format	Tool	Command	Jobs	Total Run Time
BigWig	megadepth	bash command_list.txt	1	21:26:24
BigWig	megadepth	parallel -j 4 < command_list.txt	4	05:14:28

BigWig	megadepth	parallel -j 8 < command_list.txt	8	02:45:25
BigWig	megadepth	parallel -j 16 < command_list.txt	16	01:30:39
BigWig	megadepth	parallel -j 32 < command_list.txt	32	01:05:15

*The Genotype-Tissue Expression (GTEx) Project was supported by the Common Fund of the Office of the Director of the National Institutes of Health, and by NCI, NHGRI, NHLBI, NIDA, NIMH, and NINDS. The data used for the analyses described in this manuscript were obtained from: the GTEx Portal on 11/2019 and dbGaP accession number phs000424.v8.p2 on 11/2019.

Supplementary Note S3: Comparing ability to compute sums on regions of BigWigs

Description: Compare the ability of megadepth to compute the sum of coverage on regions of BigWig files.

Data:

- Input data used was BigWig files produced from GTEx, available through recount project
- Annotations used were Gencodev35 disjoint exon and repetitive element annotation

Sub-Experiments:

Table S3a: Comparing ability to compute sum of coverage on 5 BRAIN GTEx files across annotated regions in Gencodev35 disjoint exons

Format	Tool	Command	Relative Time	Total Run Time	Average Memory (MiB)
BigWig	megadepth	megadepth <bigwig_file.bw> --annotation <bed_file.bed> --op sum > <output_file>	1.000	00:00:18	106.09
BigWig	wiggletools	wiggletools apply_paste <output_file> AUC <bed_file.bed> <bigwig_file.bw>	1.303	00:00:24	762.54
BigWig	megadepth-R	megadepth::get_coverage(bigwig_file=bw, annotation=annotation_path, op = “sum”)	2.778	00:00:50	426
BigWig	pyBigWig	python pybig_benchmark.py <bed_file.bed> <bigwig_file.bw> <output_file>	26.891	00:08:09	7.28
BigWig	bwtool	bwtool summary <bed_file.bed> <bigwigfile.bw> -with-sum -keep-bed -fill=0 -header <output_file>	35.871	00:10:52	95.78
BigWig	rtracklayer	Rscript get_coverage.R <bed_file.bed> <big_wig.bw>	45.081	00:13:40	2858.31

Table S3b: Comparing ability to compute sum of coverage on 5 BRAIN GTEx files across annotated regions in repetitive elements

Format	Tool	Command	Relative Time	Total Run Time	Average Memory (MiB)
BigWig	megadepth	megadepth <bigwig_file.bw> --annotation <bed_file.bed> --op sum > <output_file>	1.000	00:00:49	541.12
BigWig	wiggletools	wiggletools apply_paste <output_file> AUC <bed_file.bed> <bigwig_file.bw>	4.098	00:03:21	10379.14
BigWig	megadepth-R	megadepth::get_coverage(bigwig_file=bw, annotation=annotation_path, op = "sum")	2.612	00:02:08	808
BigWig	pyBigWig	python pybig_benchmark.py <bed_file.bed> <bigwig_file.bw> <output_file>	78.930	01:04:23	7.24
BigWig	bwtool	bwtool summary <bed_file.bed> <bigwigfile.bw> -with-sum -keep-bed -fill=0 -header <output_file>	103.722	01:24:36	749.68
BigWig	rtracklayer	Rscript get_coverage.R <bed_file.bed> <big_wig.bw>	105.889	01:26:22	14074.47

Table S3c: Comparing ability to compute sum of coverage on 10 BRAIN GTEx files across annotated regions in Gencodev35 disjoint exons

Format	Tool	Command	Relative Time	Total Run Time	Average Memory (MiB)
BigWig	megadepth	megadepth <bigwig_file.bw> --annotation <bed_file.bed> --op sum > <output_file>	1.000	00:00:36	101
BigWig	wiggletools	wiggletools apply_paste <output_file> AUC <bed_file.bed> <bigwig_file.bw>	1.577	00:00:57	757
BigWig	megadepth-R	megadepth::get_coverage(bigwig_file=bw, annotation=annotation_path, op = "sum")	2.778	00:01:40	426
BigWig	pyBigWig	python pybig_benchmark.py <bed_file.bed> <bigwig_file.bw> <output_file>	29.519	00:17:45	7
BigWig	bwtool	bwtool summary <bed_file.bed> <bigwigfile.bw> -with-sum -keep-bed -fill=0 -header <output_file>	38.906	00:23:24	96
BigWig	rtracklayer	Rscript get_coverage.R <bed_file.bed> <big_wig.bw>	46.962	00:28:15	2824

Table S3d: Comparing ability to compute sum of coverage on 10 BRAIN GTEx files across annotated regions in repetitive elements

Format	Tool	Command	Relative Time	Total Run Time	Average Memory (MiB)
BigWig	megadepth	megadepth <bigwig_file.bw> --annotation <bed_file.bed> --op sum > <output_file>	1.000	00:01:57	543
BigWig	wiggletools	wiggletools apply_paste <output_file> AUC <bed_file.bed> <bigwig_file.bw>	4.062	00:07:54	10379
BigWig	megadepth-R	megadepth::get_coverage(bigwig_file=bw, annotation=annotation_path, op = "sum")	2.128	00:04:09	808
BigWig	pyBigWig	python pybig_benchmark.py <bed_file.bed> <bigwig_file.bw> <output_file>	68.127	02:12:36	7
BigWig	bwtool	bwtool summary <bed_file.bed> <bigwigfile.bw> -with-sum -keep-bed -fill=0 -header <output_file>	90.480	02:56:06	750
BigWig	rtracklayer	Rscript get_coverage.R <bed_file.bed> <big_wig.bw>	100.611	03:15:49	14074

Table S3e: Summary statistics of the two annotations used in Section 5

Annotation File	Number of Regions	Min	Max	Mean	Median
Gencodev35 Disjoint Exons	665,224	1	320,039	227	98
Repetitive Elements	5,520,278	19	500,000	286	190

Supplementary Note S4: Comparing ability to output coverage per-base coverage on BAMs/CRAMs

Description: Compare the ability of megadepth to output the per-base coverage of BAM/CRAM files with other tools that have that same functionality. This mode will produce coverage across a whole genome or transcriptome depending on the input.

Data:

- Genomic BAM from Supplementary Note S1 (ERR1019041)
- RNA-seq BAM:

    - Geuvadis RNA-seq BAM (Lappalainen 2013), ERR204876_ERP001942_hg38.sorted.bam
    - For reference, this BAM is 716 MiB
- CRAM File from 1KGP (Auton et al., 2015):
    - ERR3239958
    - For reference, this CRAM is 14 GB

Sub-Experiments:

Table S4a: Comparing ability to compute per-base coverage on Genomic BAM file with single thread (ERR1019041)

Format	Tool	Command	Relative Time	Run Time	Memory (MiB)
BAM	megadepth	megadepth <bam_file.bam> --gzip --no-coverage-stdout --coverage --prefix <ouput_prefix>	1.000	00:16:59	949
BAM	mosdepth	mosdepth -F 260 <output_prefix> <bam_file.bam>	1.156	00:19:38	1904
BAM	samtools	samtools depth -a -G 260 -o <output_file_name> <bam_file.bam>	3.333	00:56:37	10
BAM	sambamba	sambamba depth base -o <output_file> -c 0 <bam_file.bam>	6.518	01:50:44	221
BAM	bedtools	bedtools genomecov -d -split -ibam <bam_file.bam> > <output_file>	7.484	02:07:09	1858
BAM	wiggletools	wiggletools coverage <bam_file.bam>	1.444	00:24:31	36

Table S4b: Comparing ability to compute per-base coverage on RNA-seq BAM file with single thread (ERR204876_ERP001942)

Format	Tool	Command	Relative Time	Run Time	Memory (MiB)
BAM	megadepth	megadepth <bam_file.bam> --gzip --no-coverage-stdout --coverage --prefix <ouput_prefix>	1.000	00:00:24	940
BAM	mosdepth	mosdepth -F 260 <output_prefix> <bam_file.bam>	2.687	00:01:05	1871
BAM	samtools	samtools depth -a -G 260 -o <output_file_name> <bam_file.bam>	41.953	00:16:52	7
BAM	sambamba	sambamba depth base -o <output_file> -c 0 <bam_file.bam>	78.216	00:31:27	51
BAM	bedtools	bedtools genomecov -d -split -ibam <bam_file.bam> > <output_file>	244.548	01:38:19	1856

BAM	wiggletools	wiggletools coverage <bam_file.bam>	1.786	00:00:43	10

Table S4c: Comparing ability to compute per-base coverage on Genomic CRAM file with single thread (ERR3239958)

Format	Tool	Command	Relative Time	Run Time	Memory (MiB)
CRAM	megadepth	megadepth <cram_file.cram> --gzip --fasta <fasta_ref> --no-coverage-stdout --coverage --prefix <ouput_prefix>	1.000	00:11:03	986
CRAM	mosdepth	mosdepth -F 260 -f <fasta_ref> <output_prefix> <cram_file.cram>	1.275	00:14:06	1912
CRAM	samtools	samtools depth -a -G 260 -o <output_file> <cram_file.cram>	4.271	00:47:13	87
CRAM	wiggletools	wiggletools coverage <cram_file.cram>	2.296	00:25:22	109

Supplementary Note S5: Comparing ability to output mean coverage on a sliding window over the genome

Description: Compare the ability of megadepth to output the average coverage on sliding windows of BAM files with other tools that have that same functionality.

Data:

- Used the Genomic BAM from Supplementary Note S1 (ERR1019041)

Sub-Experiments:

Table S5a: Comparing ability to compute mean coverage on Genomic BAM file (ERR1019041) in 500 bp partitions with single thread (run time is mean of 5 runs)

Format	Tool	Command	Relative Time	Average Run Time	Memory (MiB)
BAM	megadepth	megadepth <bam_file.bam> --gzip --op mean --annotation 500 --prefix <output_prefix> --no-annotation-stdout	1.000	00:11:33	950
BAM	mosdepth	mosdepth -F 260 -n --by 500 <output_prefix> <bam_file.bam>	1.115	00:12:53	1906
BAM	samtools	samtools -G 260 -b <bed_file> -o <output_file> <bam_file.bam>	4.973	00:57:26	105
BAM	sambamba	sambamba depth region --regions=<bed_file.bed> -o <output_file> <bam_file.bam>	5.251	01:00:39	1566
BAM	wiggletools	wiggletools apply_paste <output_file> meanI	109.657	21:06:32	5843

		<bed_file.bed> <bam_file.bam>				

Supplementary Note S6: Comparing ability to output mean coverage on regions of BAMs/CRAMs

Description: Compare the ability of megadepth to output the average coverage on given intervals of BAM and CRAM files with other tools that have that same functionality.

Data:

- Input Data:
  - Used Genomic BAM (ERR1019041) from Supplementary Note S1 as well as Genomic CRAM (ERR3239958) and RNA-seq BAM (ERR204876_ERP001942) from Supplementary Note S4
- Annotations:
  - Took Gencodev35 GTF annotation file used rtracklayer to help convert it into a GFF file with disjoint exons, then used a BEDOPs script to convert to bed file that will be used with programs.
  - Used annotation for repetitive elements.
  - Used hg19 exome annotation from Supplementary Note S1 for Simons Genomic BAM in Experiment 6a and 6b
  - Used hg38 exome annotation for 1KGP CRAM in Experiments S6g and S6h
    - Both exome annotations obtained at this link: https://www.twistbioscience.com/resources/bed-file/ngs-human-core-exome-pan el-bed-files

Sub-Experiments:

Table S6a: Comparing ability to compute mean coverage on Genomic BAM file (ERR1019041) in hg19 exome regions (1 thread)

Format	Tool	Threads	Command	Relative Time	Run Time	Memory (MiB)
BAM	megadepth	1	megadepth <bam_file.bam> --gzip --op mean --annotation <bed_file.bed> --prefix <output_prefix> --no-annotation-stdout	1.000	00:02:17	1016
BAM	mosdepth	1	mosdepth -F 260 -n --by <bed_file.bed> <output_prefix> <bam_file.bam>	5.579	00:12:43	1911
BAM	samtools	1	samtools -G 260 -b <bed_file.bed> -o <output_file> <bam_file.bam>	40.051	01:31:20	15
BAM	sambamba	1	sambamba depth region --regions=<bed_file.bed> -o <output_file> <bam_file.bam>	3.547	00:08:05	157
BAM	wiggletools	1	wiggletools apply_paste <output_file> meanI <bed_file.bed> <bam_file.bam>	628.562	23:55:13	372

Table S6b: Comparing ability to compute mean coverage on Genomic BAM file (ERR1019041) in hg19 exome regions (4 threads)

Format	Tool	Threads	Command	Relative Time	Run Time	Memory (MiB)
BAM	megadepth	4	megadepth <bam_file.bam> --threads 3 --gzip --op mean --annotation <bed_file.bed> --prefix <output_prefix> --no-annotation-stdout	1.000	00:01:05	1017
BAM	mosdepth	4	mosdepth -F 260 -n --threads 3 --by <bed_file.bed> --threads 3 <output_prefix> <bam_file.bam>	3.907	00:04:14	1913
BAM	sambamba	4	sambamba depth region --regions=<bed_file.bed> -t 4 -o <output_file> <bam_file.bam>	2.294	00:02:29	134

Table S6c: Comparing ability to compute mean coverage on RNA-seq BAM file (ERR204876_ERP001942) in Gencodev35 disjoint exon regions (1 thread)

Format	Tool	Threads	Command	Relative Time	Run Time	Memory (MiB)
BAM	megadepth	1	megadepth <bam_file.bam> --gzip --op mean --annotation <bed_file.bed> --prefix <output_prefix> --no-annotation-stdout	1.000	00:00:23	1054
BAM	mosdepth	1	mosdepth -F 260 -n --by <bed_file.bed> <output_prefix> <bam_file.bam>	2.335	00:00:53	1974
BAM	samtools	1	samtools -G 260 -b <bed_file> -o <output_file> <bam_file>	15.037	00:05:42	19
BAM	sambamba	1	sambamba depth region --regions=<bed_file.bed> -o <output_file> <bam_file.bam>	17.731	00:06:43	1011
BAM	wiggletools	1	wiggletools apply_paste <output_file> meanI <bed_file.bed> <bam_file.bam>	46.826	00:17:56	686

Table S6d: Comparing ability to compute mean coverage on RNA-seq BAM file (ERR204876_ERP001942) in Gencodev35 disjoint exon regions (4 threads)

Format	Tool	Threads	Command	Relative Time	Run Time	Memory (MiB)
BAM	megadepth	4	megadepth <bam_file.bam> --threads 3 --gzip --op mean --annotation <bed_file.bed> --prefix <output_prefix> --no-annotation-stdout	1.000	00:00:19	1055

BAM	mosdepth	4	mosdepth -F 260 -n --threads 3 --by <bed_file.bed> <output_prefix> <bam_file.bam>	2.251	00:00:44	1975
BAM	sambamba	4	sambamba depth region --regions=<bed_file.bed> -t 4 -o <output_file> <bam_file.bam>	21.211	00:06:52	1000

Table S6e: Comparing ability to compute mean coverage on RNA-seq BAM file (ERR204876_ERP001942) in repetitive element regions (1 thread)

Format	Tool	Threads	Command	Relative Time	Run Time	Memory (MiB)
BAM	megadepth	1	megadepth <bam_file.bam> --gzip --op mean --annotation <bed_file> --prefix <output_prefix> --no-annotation-stdout	1.000	00:00:39	1951
BAM	mosdepth	1	mosdepth -F 260 -n --by <bed_file.bed> <output_prefix> <bam_file.bam>	1.975	00:01:17	2442
BAM	samtools	1	samtools -G 260 -b <bed_file> -o <output_file> <bam_file>	12.639	00:08:15	92
BAM	sambamba	1	sambamba depth region --regions=<bed_file.bed> -o <output_file> <bam_file.bam>	11.230	00:07:20	1875
BAM	wiggletools	1	wiggletools apply_paste <output_file> meanI <bed_file.bed> <bam_file.bam>	13.769	00:08:57	1294

Table S6f: Comparing ability to compute mean coverage on RNA-seq BAM file (ERR204876_ERP001942) in repetitive elements regions (4 threads)

Format	Tool	Threads	Command	Relative Time	Run Time	Memory (MiB)
BAM	megadepth	4	megadepth <bam_file.bam> --threads 3 --gzip --op mean --annotation <bed_file.bed> --prefix <output_prefix> --no-annotation-stdout	1.000	00:00:35	1952
BAM	mosdepth	4	mosdepth -F 260 -n --threads 3 --by <bed_file.bed> <output_prefix> <bam_file.bam>	2.262	00:01:19	2446
BAM	sambamba	4	sambamba depth region --regions=<bed_file.bed> -t 4 -o <output_file> <bam_file.bam>	12.374	00:07:13	1842

Table S6g: Comparing ability to compute mean coverage on Genomic CRAM (ERR3239958) in hg38 exome regions (1 thread)

Format	Tool	Threads	Command	Relative Time	Run Time	Memory (MiB)
CRAM	megadepth	1	megadepth <cram_file.cram> --fasta <fasta_ref> --gzip --op mean --annotation <bed_file.bed> --prefix	1.000	00:02:16	1045

			<output_prefix> --no-annotation-stdout			
CRAM	mosdepth	1	mosdepth -F 260 -n -f <fasta_ref> --by <bed_file> <output_prefix> <cram_file.bam>	3.464	00:07:50	1926
CRAM	samtools	1	samtools -G 260 -b <bed_file> -o <output_file> <cram_file>	38.076	01:26:05	92
CRAM	wiggletools	1	wiggletools apply_paste <output_file> meanI <bed_file.bed> <cram_file.cram>	641.750	24:14:38	524

Table S6h: Comparing ability to compute mean coverage on Genomic CRAM (ERR3239958) in hg38 exome regions (4 threads)

Format	Tool	Threads	Command	Relative Time	Run Time	Memory (MiB)
CRAM	megadepth	4	megadepth <cram_file.cram> --fasta <fasta_ref> --threads 3 --gzip --op mean --annotation <bed_file.bed> --prefix <output_prefix> --no-annotation-stdout	1.000	00:01:32	1049
CRAM	mosdepth	4	mosdepth -F 260 -n --threads 3 -f <fasta_ref> --by <bed_file> <output_prefix> <cram_file.bam>	2.806	00:04:17	1993

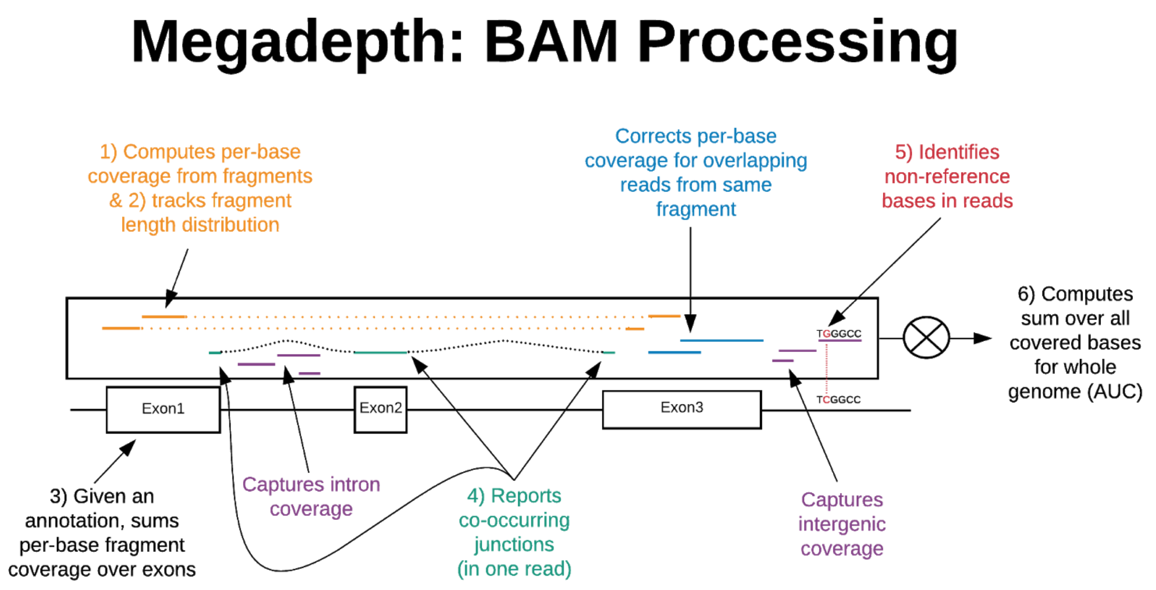

Figure S3. Coverage-related operations Megadepth can perform over a BAM file

References

Mallick, S, Li, H, Lipson, M, Mathieson, I, Gymrek, M, Racimo, F, Zhao, M, Chennagiri, N, Nordenfelt, S, Tandon, A, Skoglund, P, Lazaridis, I, Sankararaman, S, Fu, Q, Rohland, N, Renaud, G, Erlich, Y, Willems, T, Gallo, C, Spence, JP, Song, YS, Poletti, G, Balloux, F, van Driem, G, de Knijff, P, Romero, IG, Jha, AR, Behar, DM, Bravi, CM, Capelli, C, Hervig, T, Moreno-Estrada, A, Posukh, OL, Balanovska, E, Balanovsky, O, Karachanak-Yankova, S, Sahakyan, H, Toncheva, D, Yepiskoposyan, L, Tyler-Smith, C, Xue, Y, Abdullah, MS, Ruiz-Linares, A, Beall, CM, Di Rienzo, A, Jeong, C, Starikovskaya, EB, Metspalu, E, Parik, J, Villems, R, Henn, BM, Hodoglugil, U, Mahley, R, Sajantila, A, Stamatoyannopoulos, G, Wee, JT, Khusainova, R, Khusnutdinova, E, Litvinov, S, Ayodo, G, Comas, D, Hammer, MF, Kivisild, T, Klitz, W, Winkler, CA, Labuda, D, Bamshad, M, Jorde, LB, Tishkoff, SA, Watkins, WS, Metspalu, M, Dryomov, S, Sukernik, R, Singh, L, Thangaraj, K, PÃ¤Ã¤bo, S, Kelso, J, Patterson, N, Reich, D **(2016)**. The Simons Genome Diversity Project: 300 genomes from 142 diverse populations. *Nature*, 538, 7624:201-206.

Tange, O. (2011). Gnu parallel-the command-line power tool. *The USENIX Magazine*, *36*(1), 42-47.

Frankish, A, Diekhans, M, Ferreira, AM, Johnson, R, Jungreis, I, Loveland, J, Mudge, JM, Sisu, C, Wright, J, Armstrong, J, Barnes, I, Berry, A, Bignell, A, Carbonell Sala, S, Chrast, J, Cunningham, F, Di Domenico, T, Donaldson, S, Fiddes, IT, GarcÃa GirÃ³n, C, Gonzalez, JM, Grego, T, Hardy, M, Hourlier, T, Hunt, T, Izuogu, OG, Lagarde, J, Martin, FJ, MartÃnez, L, Mohanan, S, Muir, P, Navarro, FCP, Parker, A, Pei, B, Pozo, F, Ruffier, M, Schmitt, BM, Stapleton, E, Suner, MM, Sycheva, I, Uszczynska-Ratajczak, B, Xu, J, Yates, A, Zerbino, D, Zhang, Y, Aken, B, Choudhary, JS, Gerstein, M, GuigÃ³, R, Hubbard, TJP, Kellis, M, Paten, B, Reymond, A, Tress, ML, Flicek, P **(2019)**. GENCODE reference annotation for the human and mouse genomes. *Nucleic Acids Res*, 47, D1:D766-D773.

Lappalainen, T, Sammeth, M, FriedlÃ¤nder, MR, 't Hoen, PA, Monlong, J, Rivas, MA, GonzÃ lez-Porta, M, Kurbatova, N, Griebel, T, Ferreira, PG, Barann, M, Wieland, T, Greger, L, van Iterson, M, AlmlÃ¶f, J, Ribeca, P, Pulyakhina, I, Esser, D, Giger, T, Tikhonov, A, Sultan, M, Bertier, G, MacArthur, DG, Lek, M, Lizano, E, Buermans, HP, Padioleau, I, Schwarzmayr, T, Karlberg, O, Ongen, H, Kilpinen, H, Beltran, S, Gut, M, Kahlem, K, Amstislavskiy, V, Stegle, O, Pirinen, M, Montgomery, SB, Donnelly, P, McCarthy, MI, Flicek, P, Strom, TM, Lehrach, H, Schreiber, S, Sudbrak, R, Carracedo, A, Antonarakis, SE, HÃ¤sler, R, SyvÃ¤nen, AC, van Ommen, GJ, Brazma, A, Meitinger, T, Rosenstiel, P, GuigÃ³, R, Gut, IG, Estivill, X, Dermitzakis, ET, Estivill, X, Guigo, R, Dermitzakis, E, Antonarakis, S, Meitinger, T, Strom, TM, Palotie, A, Deleuze, JF, Sudbrak, R, Lerach, H, Gut, I, SyvÃ¤nen, AC, Gyllensten, U, Schreiber, S, Rosenstiel, P, Brunner, H, Veltman, J, Hoen, PA, van Ommen, GJ, Carracedo, A, Brazma, A, Flicek, P, Cambon-Thomsen, A, Mangion, J, Bentley, D, Hamosh, A **(2013)**. Transcriptome and genome sequencing uncovers functional variation in humans. *Nature*, 501, 7468:506-11.

Auton, A, Brooks, LD, Durbin, RM, Garrison, EP, Kang, HM, Korbel, JO, Marchini, JL, McCarthy, S, McVean, GA, Abecasis, GR, Auton, A, Abecasis, GR, Altshuler, DM, Durbin, RM, Abecasis, GR, Bentley, DR, Chakravarti, A, Clark, AG, Donnelly, P, Eichler, EE, Flicek, P, Gabriel, SB, Gibbs, RA, Green, ED, Hurles, ME, Knoppers, BM, Korbel, JO, Lander, ES, Lee, C, Lehrach, H, Mardis, ER, Marth, GT, McVean, GA, Nickerson, DA, Schmidt, JP, Sherry, ST, Wang, J, Wilson, RK, Gibbs, RA, Boerwinkle, E, Doddapaneni, H, Han, Y, Korchina, V, Kovar, C, Lee, S, Muzny, D, Reid, JG, Zhu, Y, Wang, J, Chang, Y, Feng, Q, Fang, X, Guo, X, Jian, M, Jiang, H, Jin, X, Lan, T, Li, G, Li, J, Li, Y, Liu, S, Liu, X, Lu, Y, Ma, X, Tang, M, Wang, B, Wang, G, Wu, H, Wu, R, Xu, X, Yin, Y, Zhang, D, Zhang, W, Zhao, J, Zhao, M, Zheng, X, Lander, ES, Altshuler, DM, Gabriel, SB, Gupta, N, Gharani, N, Toji, LH, Gerry, NP, Resch, AM, Flicek, P, Barker, J, Clarke, L, Gil, L, Hunt, SE, Kelman, G, Kulesha, E, Leinonen, R, McLaren, WM,

Radhakrishnan, R, Roa, A, Smirnov, D, Smith, RE, Streeter, I, Thormann, A, Toneva, I, Vaughan, B, Zheng-Bradley, X, Bentley, DR, Grocock, R, Humphray, S, James, T, Kingsbury, Z, Lehrach, H, Sudbrak, R, Albrecht, MW, Amstislavskiy, VS, Borodina, TA, Lienhard, M, Mertes, F, Sultan, M, Timmermann, B, Yaspo, ML, Mardis, ER, Wilson, RK, Fulton, L, Fulton, R, Sherry, ST, Ananiev, V, Belaia, Z, Beloslyudtsev, D, Bouk, N, Chen, C, Church, D, Cohen, R, Cook, C, Garner, J, Hefferon, T, Kimelman, M, Liu, C, Lopez, J, Meric, P, O'Sullivan, C, Ostapchuk, Y, Phan, L, Ponomarov, S, Schneider, V, Shekhtman, E, Sirotkin, K, Slotta, D, Zhang, H, McVean, GA, Durbin, RM, Balasubramaniam, S, Burton, J, Danecek, P, Keane, TM, Kolb-Kokocinski, A, McCarthy, S, Stalker, J, Quail, M, Durbin, RM, Balasubramaniam, S, Burton, J, Danecek, P, Keane, TM, Kolb-Kokocinski, A, McCarthy, S, Stalker, J, Quail, M, Schmidt, JP, Davies, CJ, Gollub, J, Webster, T, Wong, B, Zhan, Y, Auton, A, Campbell, CL, Kong, Y, Marcketta, A, Gibbs, RA, Yu, F, Antunes, L, Bainbridge, M, Muzny, D, Sabo, A, Huang, Z, Wang, J, Coin, LJ, Fang, L, Guo, X, Jin, X, Li, G, Li, Q, Li, Y, Li, Z, Lin, H, Liu, B, Luo, R, Shao, H, Xie, Y, Ye, C, Yu, C, Zhang, F, Zheng, H, Zhu, H, Alkan, C, Dal, E, Kahveci, F, Marth, GT, Garrison, EP, Kural, D, Lee, WP, Leong, WF, Stromberg, M, Ward, AN, Wu, J, Zhang, M, Daly, MJ, DePristo, MA, Handsaker, RE, Altshuler, DM, Banks, E, Bhatia, G, Del Angel, G, Gabriel, SB, Genovese, G, Gupta, N, Li, H, Kashin, S, Lander, ES, McCarroll, SA, Nemesh, JC, Poplin, RE, Yoon, SC, Lihm, J, Makarov, V, Clark, AG, Gottipati, S, Keinan, A, Rodriguez-Flores, JL, Korbel, JO, Rausch, T, Fritz, MH, StÃ¼tz, AM, Flicek, P, Beal, K, Clarke, L, Datta, A, Herrero, J, McLaren, WM, Ritchie, GR, Smith, RE, Zerbino, D, Zheng-Bradley, X, Sabeti, PC, Shlyakhter, I, Schaffner, SF, Vitti, J, Cooper, DN, Ball, EV, Stenson, PD, Bentley, DR, Barnes, B, Bauer, M, Cheetham, RK, Cox, A, Eberle, M, Humphray, S, Kahn, S, Murray, L, Peden, J, Shaw, R, Kenny, EE, Batzer, MA, Konkel, MK, Walker, JA, MacArthur, DG, Lek, M, Sudbrak, R, Amstislavskiy, VS, Herwig, R, Mardis, ER, Ding, L, Koboldt, DC, Larson, D, Ye, K, Gravel, S, Swaroop, A, Chew, E, Lappalainen, T, Erlich, Y, Gymrek, M, Willems, TF, Simpson, JT, Shriver, MD, Rosenfeld, JA, Bustamante, CD, Montgomery, SB, De La Vega, FM, Byrnes, JK, Carroll, AW, DeGorter, MK, Lacroute, P, Maples, BK, Martin, AR, Moreno-Estrada, A, Shringarpure, SS, Zakharia, F, Halperin, E, Baran, Y, Lee, C, Cerveira, E, Hwang, J, Malhotra, A, Plewczynski, D, Radew, K, Romanovitch, M, Zhang, C, Hyland, FC, Craig, DW, Christoforides, A, Homer, N, Izatt, T, Kurdoglu, AA, Sinari, SA, Squire, K, Sherry, ST, Xiao, C, Sebat, J, Antaki, D, Gujral, M, Noor, A, Ye, K, Burchard, EG, Hernandez, RD, Gignoux, CR, Haussler, D, Katzman, SJ, Kent, WJ, Howie, B, Ruiz-Linares, A, Dermitzakis, ET, Devine, SE, Abecasis, GR, Kang, HM, Kidd, JM, Blackwell, T, Caron, S, Chen, W, Emery, S, Fritsche, L, Fuchsberger, C, Jun, G, Li, B, Lyons, R, Scheller, C, Sidore, C, Song, S, Sliwerska, E, Taliun, D, Tan, A, Welch, R, Wing, MK, Zhan, X, Awadalla, P, Hodgkinson, A, Li, Y, Shi, X, Quitadamo, A, Lunter, G, McVean, GA, Marchini, JL, Myers, S, Churchhouse, C, Delaneau, O, Gupta-Hinch, A, Kretzschmar, W, Iqbal, Z, Mathieson, I, Menelaou, A, Rimmer, A, Xifara, DK, Oleksyk, TK, Fu, Y, Liu, X, Xiong, M, Jorde, L, Witherspoon, D, Xing, J, Eichler, EE, Browning, BL, Browning, SR, Hormozdiari, F, Sudmant, PH, Khurana, E, Durbin, RM, Hurles, ME, Tyler-Smith, C, Albers, CA, Ayub, Q, Balasubramaniam, S, Chen, Y, Colonna, V, Danecek, P, Jostins, L, Keane, TM, McCarthy, S, Walter, K, Xue, Y, Gerstein, MB, Abyzov, A, Balasubramanian, S, Chen, J, Clarke, D, Fu, Y, Harmanci, AO, Jin, M, Lee, D, Liu, J, Mu, XJ, Zhang, J, Zhang, Y, Gerstein, MB, Abyzov, A, Balasubramanian, S, Chen, J, Clarke, D, Fu, Y, Harmanci, AO, Jin, M, Lee, D, Liu, J, Mu, XJ, Zhang, J, Zhang, Y, Li, Y, Luo, R, Zhu, H, Alkan, C, Dal, E, Kahveci, F, Marth, GT, Garrison, EP, Kural, D, Lee, WP, Ward, AN, Wu, J, Zhang, M, McCarroll, SA, Handsaker, RE, Altshuler, DM, Banks, E, Del Angel, G, Genovese, G, Hartl, C, Li, H, Kashin, S, Nemesh, JC, Shakir, K, Yoon, SC, Lihm, J, Makarov, V, Degenhardt, J, Korbel, JO, Fritz, MH, Meiers, S, Raeder, B, Rausch, T, StÃ¼tz, AM, Flicek, P, Casale, FP, Clarke, L, Smith, RE, Stegle, O, Zheng-Bradley, X, Bentley, DR, Barnes, B, Cheetham, RK, Eberle, M, Humphray, S, Kahn, S, Murray, L, Shaw, R, Lameijer, EW, Batzer, MA, Konkel, MK, Walker, JA, Ding, L, Hall, I, Ye, K, Lacroute, P, Lee, C, Cerveira, E, Malhotra, A, Hwang, J, Plewczynski, D, Radew, K, Romanovitch, M, Zhang, C, Craig, DW, Homer, N, Church, D, Xiao, C, Sebat, J, Antaki, D, Bafna, V, Michaelson, J, Ye, K, Devine, SE, Gardner, EJ, Abecasis, GR, Kidd, JM, Mills, RE, Dayama, G, Emery, S, Jun, G, Shi, X, Quitadamo, A, Lunter, G, McVean, GA, Chen, K, Fan, X, Chong, Z, Chen, T,

Witherspoon, D, Xing, J, Eichler, EE, Chaisson, MJ, Hormozdiari, F, Huddleston, J, Malig, M, Nelson, BJ, Sudmant, PH, Parrish, NF, Khurana, E, Hurles, ME, Blackburne, B, Lindsay, SJ, Ning, Z, Walter, K, Zhang, Y, Gerstein, MB, Abyzov, A, Chen, J, Clarke, D, Lam, H, Mu, XJ, Sisu, C, Zhang, J, Zhang, Y, Gerstein, MB, Abyzov, A, Chen, J, Clarke, D, Lam, H, Mu, XJ, Sisu, C, Zhang, J, Zhang, Y, Gibbs, RA, Yu, F, Bainbridge, M, Challis, D, Evani, US, Kovar, C, Lu, J, Muzny, D, Nagaswamy, U, Reid, JG, Sabo, A, Yu, J, Guo, X, Li, W, Li, Y, Wu, R, Marth, GT, Garrison, EP, Leong, WF, Ward, AN, Del Angel, G, DePristo, MA, Gabriel, SB, Gupta, N, Hartl, C, Poplin, RE, Clark, AG, Rodriguez-Flores, JL, Flicek, P, Clarke, L, Smith, RE, Zheng-Bradley, X, MacArthur, DG, Mardis, ER, Fulton, R, Koboldt, DC, Gravel, S, Bustamante, CD, Craig, DW, Christoforides, A, Homer, N, Izatt, T, Sherry, ST, Xiao, C, Dermitzakis, ET, Abecasis, GR, Min Kang, H, McVean, GA, Gerstein, MB, Balasubramanian, S, Habegger, L, Gerstein, MB, Balasubramanian, S, Habegger, L, Yu, H, Flicek, P, Clarke, L, Cunningham, F, Dunham, I, Zerbino, D, Zheng-Bradley, X, Lage, K, Jespersen, JB, Horn, H, Montgomery, SB, DeGorter, MK, Khurana, E, Tyler-Smith, C, Chen, Y, Colonna, V, Xue, Y, Gerstein, MB, Balasubramanian, S, Fu, Y, Kim, D, Gerstein, MB, Balasubramanian, S, Fu, Y, Kim, D, Auton, A, Marcketta, A, Desalle, R, Narechania, A, Sayres, MA, Garrison, EP, Handsaker, RE, Kashin, S, McCarroll, SA, Rodriguez-Flores, JL, Flicek, P, Clarke, L, Zheng-Bradley, X, Erlich, Y, Gymrek, M, Willems, TF, Bustamante, CD, Mendez, FL, Poznik, GD, Underhill, PA, Lee, C, Cerveira, E, Malhotra, A, Romanovitch, M, Zhang, C, Abecasis, GR, Coin, L, Shao, H, Mittelman, D, Tyler-Smith, C, Ayub, Q, Banerjee, R, Cerezo, M, Chen, Y, Fitzgerald, TW, Louzada, S, Massaia, A, McCarthy, S, Ritchie, GR, Xue, Y, Yang, F, Tyler-Smith, C, Ayub, Q, Banerjee, R, Cerezo, M, Chen, Y, Fitzgerald, TW, Louzada, S, Massaia, A, McCarthy, S, Ritchie, GR, Xue, Y, Yang, F, Gibbs, RA, Kovar, C, Kalra, D, Hale, W, Muzny, D, Reid, JG, Wang, J, Dan, X, Guo, X, Li, G, Li, Y, Ye, C, Zheng, X, Altshuler, DM, Flicek, P, Clarke, L, Zheng-Bradley, X, Bentley, DR, Cox, A, Humphray, S, Kahn, S, Sudbrak, R, Albrecht, MW, Lienhard, M, Larson, D, Craig, DW, Izatt, T, Kurdoglu, AA, Sherry, ST, Xiao, C, Haussler, D, Abecasis, GR, McVean, GA, Durbin, RM, Balasubramaniam, S, Keane, TM, McCarthy, S, Stalker, J, Durbin, RM, Balasubramaniam, S, Keane, TM, McCarthy, S, Stalker, J, Chakravarti, A, Knoppers, BM, Abecasis, GR, Barnes, KC, Beiswanger, C, Burchard, EG, Bustamante, CD, Cai, H, Cao, H, Durbin, RM, Gerry, NP, Gharani, N, Gibbs, RA, Gignoux, CR, Gravel, S, Henn, B, Jones, D, Jorde, L, Kaye, JS, Keinan, A, Kent, A, Kerasidou, A, Li, Y, Mathias, R, McVean, GA, Moreno-Estrada, A, Ossorio, PN, Parker, M, Resch, AM, Rotimi, CN, Royal, CD, Sandoval, K, Su, Y, Sudbrak, R, Tian, Z, Tishkoff, S, Toji, LH, Tyler-Smith, C, Via, M, Wang, Y, Yang, H, Yang, L, Zhu, J, Bodmer, W, Bedoya, G, Ruiz-Linares, A, Cai, Z, Gao, Y, Chu, J, Peltonen, L, Garcia-Montero, A, Orfao, A, Dutil, J, Martinez-Cruzado, JC, Oleksyk, TK, Barnes, KC, Mathias, RA, Hennis, A, Watson, H, McKenzie, C, Qadri, F, LaRocque, R, Sabeti, PC, Zhu, J, Deng, X, Sabeti, PC, Asogun, D, Folarin, O, Happi, C, Omoniwa, O, Stremlau, M, Tariyal, R, Jallow, M, Joof, FS, Corrah, T, Rockett, K, Kwiatkowski, D, Kooner, J, HiÃªn, TT, Dunstan, SJ, Hang, NT, Fonnie, R, Garry, R, Kanneh, L, Moses, L, Sabeti, PC, Schieffelin, J, Grant, DS, Gallo, C, Poletti, G, Saleheen, D, Rasheed, A, Saleheen, D, Rasheed, A, Brooks, LD, Felsenfeld, AL, McEwen, JE, Vaydylevich, Y, Green, ED, Duncanson, A, Dunn, M, Schloss, JA, Wang, J, Yang, H, Auton, A, Brooks, LD, Durbin, RM, Garrison, EP, Min Kang, H, Korbel, JO, Marchini, JL, McCarthy, S, McVean, GA, Abecasis, GR, Auton, A, Brooks, LD, Durbin, RM, Garrison, EP, Min Kang, H, Korbel, JO, Marchini, JL, McCarthy, S, McVean, GA, Abecasis, GR **(2015)**. A global reference for human genetic variation. *Nature*, 526, 7571:68-74.
